# Orthopoxvirus Circulation in an Endemic Area in Brazil: Investigation of Infections in Small Mammals during an Absence of Outbreaks

**DOI:** 10.3390/v15040842

**Published:** 2023-03-25

**Authors:** Iago J. S. Domingos, Kamila L. S. Rocha, Jessica M. Graciano, Lara R. Almeida, Jeffrey B. Doty, Adriano P. Paglia, Danilo B. Oliveira, Yoshinori J. Nakazawa, Giliane de S. Trindade

**Affiliations:** 1 Instituto de Ciências Biológicas, Universidade Federal de Minas Gerais, Avenida Antonio Carlos, 6627, Belo Horizonte 31270-901, Brazil; 2 U.S. Centers for Disease Control and Prevention, Poxvirus and Rabies Branch, 1600 Clifton Rd. NE, Atlanta, GA 30333, USA; 3 Centro Integrado de Pesquisa e Pós-Graduação, Universidade Federal dos Vales do Jequitinhonha e Mucuri, MGC 367 Km 583, 5000, Diamantina 39100-000, Brazil

**Keywords:** *Vaccinia virus*, epidemiology, animal diseases, public health, ecology

## Abstract

Vaccinia virus (VACV) is the causative agent of an emerging viral zoonosis called bovine vaccinia (BV). Several studies have documented characteristics of VACV infections in Brazil; however, the manner in which this virus is maintained in wildlife remains unknown. This work investigated the presence of viral DNA and anti-orthopoxvirus (OPXV) antibodies in samples collected from small mammals in a VACV-endemic area in Minas Gerais, Brazil, in the absence of current outbreaks. Samples did not show amplification of OPXV DNA in molecular tests. However, 5/142 serum samples demonstrated the presence of anti-OPXV neutralizing antibodies in serological tests. These data reinforce the involvement of small mammals in the natural cycle of VACV, highlighting the need for further ecological studies to better understand how this virus is maintained in nature and to develop measures to prevent BV outbreaks.

## 1. Introduction

Infectious diseases caused by viruses belonging to the *Orthopoxvirus* (OPXV) genus have been present among humans for thousands of years, including smallpox, caused by variola virus (VARV), which has been responsible for millions of deaths throughout human history [1]. The eradication of smallpox occurred thanks to other OPXVs, cowpox virus (CPXV) and later vaccinia virus (VACV), the latter was used in worldwide vaccination programs promoted by the World Health Organization [1,2]. Following the eradication of smallpox in 1980, infections by zoonotic OPXVs have emerged among humans in diverse geographic locations, such as CPXV infections in Europe, monkeypox virus (MPXV) historically mostly in Central and West Africa, and VACV, primarily in Brazil in South America [3,4,5,6].

Vaccinia virus was first described to cause natural infections in Brazil at the end of the 20th century and has since been associated with outbreaks of bovine vaccinia (BV), a disease affecting dairy cattle and milkers. In cattle, this disease is typically characterized by vesiculopustular exanthematic lesions on the udders and teats of milking cows. In humans, the lesions appear mostly on the hands of milkers which can lead to secondary infections, but systemic symptoms such as fever, myalgia, and headache are also common [7,8,9]. VACV is widely distributed in Brazil, and over the last twenty years, several characteristics of the virus and the disease it causes have been investigated and described [10,11]. The virus has been detected from several sources, such as lesion material, animal excrements, blood, and milk and its derivatives, and previous studies have documented factors that have contributed to VACV spreading throughout the country [10,11,12,13,14]. Additionally, a wide range of hosts has been reported for this virus, such as humans, bovines, equines, and small mammals [6,10,12,13,14]. Viral DNA, viral isolates, and anti-OPXV neutralizing antibodies have been successfully detected in field and laboratory-based studies [15,16,17,18]. These reports filled important gaps in VACV epidemiology and provided insight about the circulation of this virus in animal hosts across the country. However, the natural reservoir(s) and maintenance of VACV in nature remain poorly understood [9,10,11].

Small mammals have been implicated as reservoirs for other OPXVs; serological and molecular studies have shown that three rodent species, *Apodemus sylvaticus*, *Microtus agrestis,* and *Myodes glareolus,* are involved in the natural circulation of CPXV [19]. Similarly, several small mammal species have been suggested as putative reservoirs of MPXV, including African rodents of the genera *Funisciurus*, *Cricetomys,* and *Graphiurus*, among others, but more studies are necessary to better describe the circulation of this virus in nature [20,21,22].

Beyond CPXV and MPXV, an extensive range of studies conducted in Brazilian natural and rural areas have detected evidence of VACV infection in several small mammal species (Rodentia and Didelphimorphia) using molecular and serological tests [13,15,16,17,23,24]. Viable VACV was detected in *Mus musculus* during a bovine vaccinia outbreak investigation [24]. In addition, anti-OPXV neutralizing antibodies have been detected in blood samples from naturally infected rodents and marsupials [16,25]. Viral isolates have also been obtained from rodent feces and urine [13]. These studies support the inclusion of small mammals in the host range of VACV and could contribute to the spread of the virus between different environments.

Serro county (18°36′21″ S, 43°23′13″ W) is located in an area of Brazil where VACV is known to be endemic and has recorded several BV outbreaks, with OPXV infections detected in bovines, equids, rodents, marsupials, and humans [14,16,26,27,28]. The city is located in Vale do Jequitinhonha, 329 km northeast from Belo Horizonte, the capital of Minas Gerais state [29,30]. Serro is in a transition area between Cerrado and Atlantic Forest in Brazil, and these biomes confer unique characteristics to the region. The savanna-like Cerrado biome is the second largest in the country and exhibits a large biodiversity with a diverse number of animal species [31,32]. Atlantic Forest is also an important biome in Brazil where a high diversity of rodents and marsupial species can be found and has been suffering from degradation for thousands of years [32]. The city of Serro is a well-known city in the country due to its traditional production of artisanal cheese from raw milk, which is a very important component in its economy [30]. Due to the potential harms VACV infections could cause to this industry, studies to understand VACV circulation in this region are needed.

The present work aims to detect evidence of the circulation of OPXV in small mammals captured in and around dairy farms producing artisanal cheese in the city of Serro-MG, Brazil. The study included the capture and sampling of wild animals, testing of liver and serum samples through molecular (quantitative polymerase chain reaction amplification—qPCR), and serological (plaque reduction neutralization test—PRNT) tests, respectively. Through this study, we aim to contribute to the understanding of VACV circulation in wild animals found in and around farms and how the virus is maintained in the environment. In addition, the results from this study may inform VACV prevention and control efforts in dairy farms by identifying animal species potentially involved in the circulation of this virus.

## 2. Materials and Methods

### 2.1. Small Mammal Sampling Procedures

**Figure 1:**
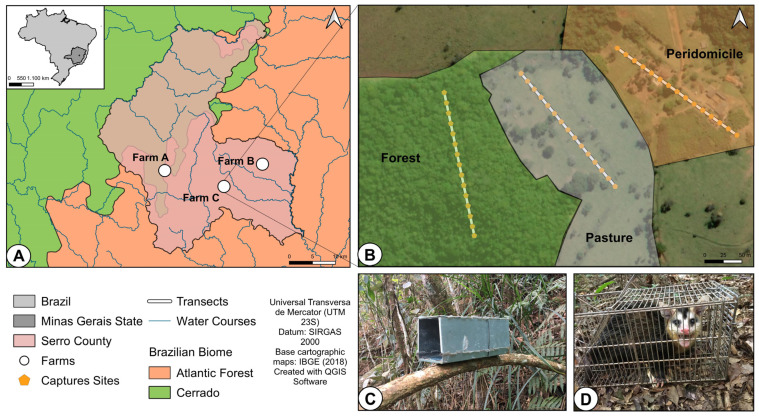
Geographic location, sampling design, and traps used during the study in Serro-MG, Brazil. (**A**) Map of Serro county (pink), water courses are blue lines and the Brazilian biomes intersecting in this region are depicted: Cerrado Biome in green and Atlantic Forest Biome in orange. Map inset at the top left shows the location of the State of Minas Gerais. Farms selected for the study are indicated by white circles. (**B**) Sampling design in one of the farms, showing three transects (white lines), with the fifteen capture sites each (yellow pentagons). (**C**) Sherman live-trap positioned on a tree branch. (**D**) Tomahawk-like cage positioned on the ground with a common opossum (*Didelphis aurita*) captured.

A total of three farms were chosen in Serro county (Figure 1). The farms had an average distance of 14 km from each other. At each farm, three habitat types (peridomicile, pasture, and forest) were selected, and one sampling transect was established in each habitat. Each transect consisted of 15 capture sites every 15 m; capture sites had two live-traps: a Tomahawk-like trap (25 × 25 × 40 cm) and a Sherman live-trap (9 × 9 × 22 cm) for a total of 30 traps per transect. Therefore, each farm had 45 capture sites and 90 traps for a total of 270 traps across the three farms. All traps were checked every day for 14 consecutive days during four field expeditions distributed in February, May, July, and August of 2021. The total sampling effort was 15,120 traps-nights. The baits used were a small cotton ball soaked in liver fish oil and a banana chunk, and these were switched on alternate days. Captured animals were transported alive to the local laboratory for sample collection.

During animal manipulation, personal protective equipment was used as recommended by the U.S. Centers for Disease Control and Prevention (CDC) [33]. All procedures with animals were authorized by the Ethics Commission on Animal Use (CEUA) of the Federal University of Minas Gerais under Protocol No. 217/2020 and the CDC Institutional Animal Care and Use Committee (IACUC) under protocol 3183DOTMULX. Small mammal sample collection was carried out under ICMBio Authorization No. 129894.

A veterinarian was responsible for anesthesia administration and euthanasia procedures for all captured animals. Animals were anesthetized with a combination of ketamine (70 mg/kg) and xylazine (10 mg/kg) prior to blood collection and euthanasia. Data were recorded from each animal including taxonomy, standard measurements, sex, and relative age (juvenile vs. adult). Marsupials belonging to the genus *Didelphis* and pregnant animals were not euthanized and were released at the site of capture following blood and data collection.

Blood samples were centrifuged at 8000 rcf for two minutes to separate the serum from the clot. Gonads, bladder, intestines, stomach, kidneys, spleen, liver, lungs, and heart were collected from each euthanized animal, and urine and feces samples were collected when present. Liver and serum samples were selected for OPXV screening, and if viral amplification was observed, additional sample types would be tested. Each tissue sample was collected in duplicate and stored in two different microtubes: a dry cryogenic microtube and an Eppendorf microtube with 400 μL of RNA Later (Invitrogen, Waltham, MA, USA) solution. Samples in cryogenic microtubes were stored in liquid nitrogen, while the RNA Later samples were stored in −20 °C freezers until being transported to the BSL-2 laboratory in Diamantina city, Minas Gerais, Brazil, where all the samples were stored in a −80 °C freezer until analysis.

### 2.2. Laboratory Analysis

DNA was extracted using the PureLink Genomic DNA Mini Kit (Invitrogen, Waltham, MA, USA) following the manufacturer instructions, and the samples were not homogenized or processed prior to extraction. Serum diluted to 1:10 was used for real-time PCR without DNA extraction, as previously described [34]. Serum and liver DNA samples were tested for three different OPXV gene targets: the C11R gene [34], the A56R gene [35], and the E9L gene [36], which encode the virus growth factor (VGF), the viral hemagglutinin (HA), and the viral DNA polymerase, respectively (Table S1). The mouse β-actin gene was used as an endogenous control and a VACV Western Reserve strain (Laboratório de Vírus, UFMG, Belo Horizonte, Brazil) was used as a positive control. All three targets were tested in duplicate with a 10 μL final reaction volume using the StepOne^®^ Applied Biosystems software version 2.3. The C11R and A56R assays used SYBR^®^ Green I Master Mix with these cycling conditions: denaturation at 95 °C/20 min, 40 cycles of 95 °C/3 s and 60 °C/20 s, and a melting curve using 95 °C/3 s and 60 °C/20 s, followed by an increase in temperature of 4 °C until reaching 95 °C/15 s. The E9L gene assay used TaqMan^®^ Master Mix with cycling conditions of 50 °C/2 min and 95 °C/10 s, followed by 40 cycles of 95 °C/15 s and 60 °C/1 min.

In order to detect and quantify anti-OPXV neutralizing antibodies, all serum samples were analyzed by PRNT as previously described [37] and included previously published modifications [34]. BSC-40 cells for PRNT assays were cultivated in Eagle’s minimum essential medium (MEM) supplemented with 5% fetal bovine serum (FBS), 1% amphotericin, 1% penicillin-streptomycin solution, and 1% L-glutamine. A VACV Western Reserve strain was utilized as a positive control. The cytopathic effect in BSC-40 cells was analyzed and the samples were considered positive if at least a 50% reduction was observed when compared with the virus control plates, and the neutralizing titer per mL (IU/mL) of these samples was determined considering the final 1:40 dilution.

## 3. Results

### 3.1. Animal Collection

At the end of the four expeditions, a total of 210 small mammals were captured, with a capture success of 1.39%. Of these, 143 (68.10%) belonged to 7 species of the order Didelphimorphia, and 67 (31.90%) belonged to 15 species of the order Rodentia (Table S2). Liver was collected from 107 animals (50.95% of the total number of captures): 46 (42.99%) from marsupials and 61 (57.01%) from rodents. Serum was collected from 142 (67.62%) animals: 88 (61.97%) from marsupials and 54 (38.03%) from rodents. Tomahawk traps captured 148 animals (70.48%), while Sherman traps yielded 62 captures (29.52%). The sex of the captured animals was equally distributed, with 92 (43.81%) females, 92 (43.81%) males, and 26 (12.38%) unidentified. For the relative age, 123 (58.57%) animals were adult, 45 (21.43%) were juvenile, 3 (1.43%) were infant, and 39 (18.57%) were not possible to define. The forest was the most successful habitat type with 119 (56.67%) captures, followed by peridomicile with 63 (30%) captures, and pasture with 28 (13.33%) captures.

### 3.2. Laboratory Findings

**Figure 2:**
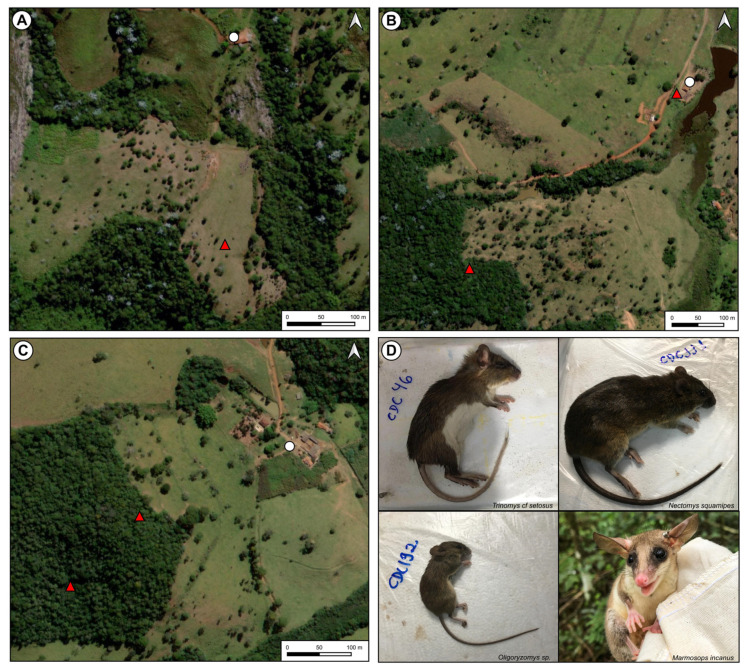
Capture location of seropositive species, Brazil, 2021. The (**A**–**C**) farm buildings are indicated by white circles and spots of seropositive animal capture by red triangles. *Trinomys cf setosus, Nectomys squamipes,* and *Oligoryzomys* sp. in panel (**D**) were photographed after anesthesia administration. *Marmosops incanus* in panel (**D**) was photographed after its capture in the field.

While amplification was detected for the mouse β-actin gene in all samples and in all PCR positive control samples, none of the liver or serum samples showed amplification of any of the three OPXV gene targets, indicating that no viral DNA was present. Among the 142 serum samples tested, 5 (3.52%) showed the presence of anti-OPXV neutralizing antibodies in the PRNTs. All positive animals were captured during dry seasons but from three different field expeditions. Neutralizing antibodies were detected in samples from three rodent species (*Trinomys cf setosus, Nectomys squamipes,* and *Oligoryzomys* sp.) and from two animals of one marsupial species (*Marmosops incanus*) (Figure 2). At least one seropositive animal was captured from each farm and each habitat type (Table 1).

## 4. Discussion

Zoonotic diseases have major impacts on public health and small mammals have been directly related to their maintenance in nature and in transmission to people [38]. Rodents are one of the primary reservoirs for zoonotic pathogens, which indicates a need for intense surveillance efforts in order to prevent and mitigate the risks of zoonotic infections in human populations [39,40]. This includes OPXVs, some of which are associated with small mammals and have previously been shown to be very harmful to human health [41,42,43]. Identifying the role of rodents and marsupials in the spread and maintenance of VACV will allow the development of prevention strategies and improve public health preparedness.

The diversity of animals sampled in this study from the different sampling sessions is in line with the documented diversity of Cerrado and Atlantic Forest in Brazil [44,45,46]. During the first sampling session in February of 2021, the weather conditions were very rainy and hot—as usually seen during summer in Brazil. The next session occurred in May of 2021 during autumn, a dry and cold season. The third and fourth sessions, in July and August of 2021, respectively, occurred during winter, when the weather was dryer and colder than autumn. All these characteristics affect small mammal behavior; for example, during dry seasons it is common to observe low availability of food for small mammals, which can favor the abundance of these animals in peridomicile areas due to increased foraging [47]. Additionally, in many small mammal species, it is more likely to see adult animals foraging than juvenile animals [46], which may have contributed to the high proportion of adult animals captured in this study.

Liver samples spiked with VACV-WR as positive DNA extraction controls showed no inhibition for extraction or PCR. In addition, β-actin gene and positive control amplification in all PCR tests indicated the assays were performed as expected. In the present study, it was not possible to detect viral DNA in the tested samples. However, anti-OPXV neutralizing antibodies were detected in a small percentage of serum samples from rodents and marsupials collected from different habitats, different farms, and different months of the year. The negative results both in PCR tests and in PRNT assays suggest that most animals sampled had no exposure to VACV. In order to explain these results, it is important to consider the absence of VACV outbreaks in Serro in recent years, as this might be one of the causes of the low seroprevalence in the small mammals sampled in this study. Additionally, the difficulty of sampling an animal with an active infection likely impacted the lack of molecular detection. When a VACV infection occurs in small mammals, similar to what is usually observed in humans, it is unlikely that the animal stays persistently infected for a long period of time [11,26,27]. After the animal recovers, it is not expected to exhibit detectable VACV or viral DNA in samples, which highlights the low probability of sampling an infected animal.

The small number of PRNT-positive animals shows the presence of anti-OPXV neutralizing antibodies at low levels in the mammal community. The anti-OPXV neutralizing antibodies were detected in samples collected in the three different habitat types, suggesting the circulation of VACV in each of these areas. Previous studies have shown hypothetical routes of VACV circulation among small mammals found among farms, disturbed areas near farms, and forest areas [16,24]. The seropositive results in this study support these findings, reinforcing the need for additional studies to describe the zoonotic VACV cycle. Evidence of VACV infections in *Trinomys cf setosus* and *Nectomys squamipes* have been previously reported from Serro county, while *Oligoryzomys* sp. have been associated with VACV in other locations in Brazil [16,25]. To our knowledge, this is the first report of anti-OPXV neutralizing antibodies identified in *Marmosops incanus* samples. Interestingly, this animal is a semi-arboreal marsupial with low locomotory behavior and is commonly found in forest areas [48]. The seropositivity in these animals shows the need for additional research into the spread of VACV to new species.

Previous studies have suggested an epidemiological chain between wildlife and domestic animals, with small mammals playing a pivotal role in this cycle [18,23,25]. However, the mechanisms of viral circulation remain a mystery. The results presented here provide additional insight into how VACV is maintained in endemic regions, as the positive serological results demonstrate that the virus is silently circulating in the environment. Ultimately, more longitudinal surveillance studies are needed to examine the natural history of VACV in Brazil.

## Figures and Tables

**Table 1:** Captured animals’ data with positive results in PRNT assays.

Sample	Capture Date	Weather Conditions	Order	Genus/Species	Sex	Age	Farm	Site of Capture	PRNT *			PCR **		
									Result	Plaque Reduction Percentage	Neutralizing Units per mL	C11R Gene	A56R Gene	E9L Gene
46	11 May 2021	Dry	Rodentia	*Trinomys cf setosus*	Female	Adult	B	Forest	+	63.51%	100 NU/mL	-	-	-
84	1 July 2021	Dry	Didelphimorphia	*Marmosops incanus*	Male	Adult	C	Forest	+	70.54%	100 NU/mL	-	-	-
111	7 July 2021	Dry	Rodentia	*Nectomys squamipes*	Male	Adult	B	Peridomicile	+	84.48%	100 NU/mL	-	-	-
142	3 August 2021	Dry	Didelphimorphia	*Marmosops incanus*	Male	Adult	C	Forest	+	60.00%	100 NU/mL	-	-	-
192	14 August 2021	Dry	Rodentia	*Oligoryzomys* sp.	Unidentifed	Juvenile	A	Pasture	+	55.45%	100 NU/mL	-	-	-

* All PRNT assays were performed from serum samples. The plaque reduction percentage and the neutralizing units per mL values represent the results observed in duplicate serum samples. NU/mL: neutralizing units per mL. ** All three PCR gene targets (C11R, A56R, and E9L) were tested in both liver and serum samples from the animals.

## Data Availability

The data presented in this study are available within the article and in the Supplementary Material.

## Supplementary Materials

The following supporting information can be downloaded at: https://www.mdpi.com/article/10.3390/v15040842/s1, Table S1: Primers and probe sequences from OPV genes C11R, A56R and E9L; Table S2: Small mammals’ data from field expeditions.
