# Supplementary material for: Orthopoxvirus Circulation in an Endemic Area in Brazil: Investigation of Infections in Small Mammals during an Absence of Outbreaks
Source: Viruses. 2023 Mar 25;15(4):842. doi: 10.3390/v15040842 (PMC10144947; doi:10.3390/v15040842)
Supplement: Supplementary file 1 [file viruses-15-00842-s001.zip › Table S1.pdf]

Orthopoxvirus circulation in an endemic area in Brazil: Investigation of infections in small mammals during an absence of outbreaks

Table S1. Primers and probe sequences from OPV genes C11R, A56R and E9L

Gene Primers and Probe	Sequence	Reference
C11R Forward	5′ CGCTACAACAGATATTCCAGCTATCAG 3′	*34*
C11R Reverse	5′ AGCGTGGATACAGTCACCGTGTAA 3′	
A56R Forward	5′ CATCATCTGGAATTGTCACTACTAAA 3′	*35*
A56R Reverse	5′ ACGGCCGACAATATAATTAATGC 3′	
E9L Forward	5′ TCAACTGAAAAGGCCATCTATGA 3′	*36*
E9L Reverse	5′ GAGTATAGAGCACTATTTCTAAATCCCA 3′	
E9L-NVAR probe	5′ TET-CCATGCAATATACGTACAAGATAGTAGCCAAC 3′	
