# Supplementary material for: Orthopoxvirus Circulation in an Endemic Area in Brazil: Investigation of Infections in Small Mammals during an Absence of Outbreaks
Source: Viruses. 2023 Mar 25;15(4):842. doi: 10.3390/v15040842 (PMC10144947; doi:10.3390/v15040842)
Supplement: Supplementary file 1 [file viruses-15-00842-s001.zip › Table S2.pdf]

Orthopoxvirus circulation in an endemic area in Brazil: Investigation of infections in small mammals during an absence of outbreaks

Table S2. Small mammals data from field expeditions

Order	Genus/Species	Number of Captures		Samples				Site of capture						Weather conditions				Farms					
				Liver		Serum		Forest		Pasture		Peridomicile		Dry		Rainy		A		B		C	
		no.	(%)	no.	(%)	no.	(%)	no.	(%)	no.	(%)	no.	(%)	no.	(%)	no.	(%)	no.	(%)	no.	(%)	no.	(%)
Didelphimorphia	*Caluromys philander*	1	(0.48)	1	(0.93)	1	(0.70)	1	(0.84)	-	-	-	-	1	(0.63)	-	-	1	(2.70)	-	-	-	-
	Didelphis albiventris	29	(13.81)	1	(0.93)	16	(11.27)	26	(21.85)	3	(10.71)	-	-	25	(15.72)	4	(7.84)	-	-	24	(30.77)	5	(5.26)
	Didelphis aurita	67	(31.90)	3	(2.80)	30	(21.13)	40	(33.61)	11	(39.29)	16	(25.40)	58	(36.48)	9	(17.65)	4	(10.81)	18	(23.08)	45	(47.37)
	Gracilianus sp.	2	(0.95)	2	(1.87)	2	(1.41)	1	(0.84)	-	-	1	(1.59)	2	(1.26)	-	-	2	(5.41)	-	-	-	-
	Marmosops incanus	40	(19.05)	35	(32.71)	36	(25.35)	37	(31.09)	-	-	3	(4.76)	28	(17.61)	12	(23.53)	7	(18.92)	33	(42.31)	-	-
	Monodelphis americana	1	(0.48)	1	(0.93)	-	-	1	(0.84)	-	-	-	-	1	(0.63)	-	-	-	-	1	(1.28)	-	-
	Philander frenatus	3	(1.43)	3	(2.80)	3	(2.11)	1	(0.84)	-	-	2	(3.17)	2	(1.26)	1	(1.96)	3	(8.11)	-	-	-	-
	Total of marsupials	143	(68.10)	46	(42.99)	88	(61.97)	107	(89.92)	14	(50.00)	22	(34.92)	117	(73.58)	26	(50.98)	17	(45.95)	76	(97.44)	50	(52.63)
Rodentia	*Akodon sp.*	1	(0.48)	1	(0.93)	1	(0.70)	1	(0.84)	-	-	-	-	1	(0.63)	-	-	1	(2.70)	-	-	-	-
	Calomys cf tener	2	(0.95)	1	(0.93)	-	-	-	-	2	(7.14)	-	-	2	(1.26)	-	-	1	(2.70)	1	(1.28)	-	-
	Calomys cf expulsus	3	(1.43)	3	(2.80)	3	(2.11)	-	-	3	(10.71)	-	-	3	(1.89)	-	-	-	-	-	-	3	(3.16)
	Calomys sp.	6	(2.86)	6	(5.61)	6	(4.23)	-	-	1	(3.57)	5	(7.94)	1	(0.63)	5	(9.80)	1	(2.70)	-	-	5	(5.26)
	Cerradomys sp.	1	(0.48)	1	(0.93)	1	(0.70)	1	(0.84)	-	-	-	-	1	(0.63)	-	-	1	(2.70)	-	-	-	-
	Cerradomys subflavus	1	(0.48)	1	(0.93)	1	(0.70)	-	-	-	-	1	(1.59)	1	(0.63)	-	-	1	(2.70)	-	-	-	-
	Mus musculus	7	(3.33)	5	(4.67)	4	(2.82)	-	-	-	-	7	(11.11)	2	(1.26)	5	(9.80)	-	-	-	-	7	(7.37)
	Necromys lasiurus	18	(8.57)	15	(14.02)	13	(9.15)	-	-	4	(14.29)	14	(22.22)	8	(5.03)	10	(19.61)	1	(2.70)	-	-	17	(17.89)
	Necromys sp.	1	(0.48)	1	(0.93)	1	(0.70)	-	-	-	-	1	(1.59)	1	(0.63)	-	-	-	-	-	-	1	(1.05)
	Nectomys squamipes	1	(0.48)	1	(0.93)	1	(0.70)	-	-	-	-	1	(1.59)	1	(0.63)	-	-	-	-	-	-	1	(1.05)
	Oligoryzomys nigripes	8	(3.81)	8	(7.48)	8	(5.63)	4	(3.36)	1	(3.57)	3	(4.76)	6	(3.77)	2	(3.92)	2	(5.41)	1	(1.28)	5	(5.26)
	Oligoryzomys sp.	11	(5.24)	11	(10.28)	8	(5.63)	2	(1.68)	3	(10.71)	6	(9.52)	9	(5.66)	2	(3.92)	8	(21.62)	-	-	3	(3.16)
	Rattus rattus	3	(1.43)	3	(2.80)	3	(2.11)	-	-	-	-	3	(4.76)	3	(1.89)	-	-	2	(5.41)	-	-	1	(1.05)
	Rhipidomys sp.	2	(0.95)	2	(1.87)	2	(1.41)	2	(1.68)	-	-	-	-	2	(1.26)	-	-	1	(2.70)	-	-	1	(1.05)
	Trinomys cf setosus	2	(0.95)	2	(1.87)	2	(1.41)	2	(1.68)	-	-	-	-	1	(0.63)	1	(1.96)	1	(2.70)	-	-	1	(1.05)
	Total of rodents	67	(31.90)	61	(57.01)	54	(38.03)	12	(10.08)	14	(50.00)	41	(65.08)	42	(26.42)	25	(49.02)	20	(54.05)	2	(2.56)	45	(47.37)
Total of samples		210	(100.00)	107	(100.00)	142	(100.00)	119	(100.00)	28	(100.00)	63	(100.00)	159	(100.00)	51	(100.00)	37	(100.00)	78	(100.00)	95	(100.00)
